# Assessment of decision-making with locally run and web-based large language models versus human board recommendations in otorhinolaryngology, head and neck surgery

**DOI:** 10.1007/s00405-024-09153-3

**Published:** 2025-01-10

**Authors:** Christoph Raphael Buhr, Benjamin Philipp Ernst, Andrew Blaikie, Harry Smith, Tom Kelsey, Christoph Matthias, Maximilian Fleischmann, Florian Jungmann, Jürgen Alt, Christian Brandts, Peer W. Kämmerer, Sebastian Foersch, Sebastian Kuhn, Jonas Eckrich

**Affiliations:** 1 Department of Otorhinolaryngology, University Medical Center of the Johannes Gutenberg-University Mainz, Langenbeckstraße 1, 55131 Mainz, Germany; 2 School of Medicine, University of St Andrews, St Andrews, UK; 3 Department of Otorhinolaryngology, University Medical Center Frankfurt, Theodor-Stern-Kai 7, 60596 Frankfurt am Main, Germany; 4 School of Computer Science, University of St Andrews, St Andrews, UK; 5 Department of Radiotherapy and Oncology, University Medical Center Frankfurt, Theodor-Stern-Kai 7, 60596 Frankfurt am Main, Germany; 6 Outpatient Department for Radiology and Nuclear Medicine at Marienhaus Hospital Saarlouis, Xcare Group, Kapuzinerstraße 4, 66740 Saarlouis, Germany; 7 Department of Hematology and Medical Oncology, University Medical Center Mainz of the Johannes Gutenberg-University Mainz, Langenbeckstraße 1, 55131 Mainz, Germany; 8 Department of Hematology and Medical Oncology, University Medical Center Frankfurt, Theodor-Stern-Kai 7, 60596 Frankfurt am Main, Germany; 9 Department of Oral and Maxillofacial Surgery—Plastic Surgery, University Medical Center Mainz of the Johannes Gutenberg-University Mainz, Langenbeckstraße 1, 55131 Mainz, Germany; 10 Institute of Pathology, University Medical Center of the Johannes Gutenberg-University Mainz, Langenbeckstraße 1, 55131 Mainz, Germany; 11 Institute for Digital Medicine, Philipps-University Marburg and University Hospital of Giessen and Marburg, Marburg, Germany

**Keywords:** Large language models, LLM, Artificial intelligence, AI, ChatGPT, Llama, Otorhinolaryngology, ORL, Head and neck, Digital health, Chatbot, Language model

## Abstract

**Introduction:**

Tumor boards are a cornerstone of modern cancer treatment. Given their advanced capabilities, the role of Large Language Models (LLMs) in generating tumor board decisions for otorhinolaryngology (ORL) head and neck surgery is gaining increasing attention. However, concerns over data protection and the use of confidential patient information in web-based LLMs have restricted their widespread adoption and hindered the exploration of their full potential. In this first study of its kind we compared standard human multidisciplinary tumor board recommendations (MDT) against a web-based LLM (ChatGPT-4o) and a locally run LLM (Llama 3) addressing data protection concerns.

**Material and methods:**

Twenty-five simulated tumor board cases were presented to an MDT composed of specialists from otorhinolaryngology, craniomaxillofacial surgery, medical oncology, radiology, radiation oncology, and pathology. This multidisciplinary team provided a comprehensive analysis of the cases. The same cases were input into ChatGPT-4o and Llama 3 using structured prompts, and the concordance between the LLMs' and MDT’s recommendations was assessed. Four MDT members evaluated the LLMs' recommendations in terms of medical adequacy (using a six-point Likert scale) and whether the information provided could have influenced the MDT's original recommendations.

**Results:**

ChatGPT-4o showed 84% concordance (21 out of 25 cases) and Llama 3 demonstrated 92% concordance (23 out of 25 cases) with the MDT in distinguishing between curative and palliative treatment strategies. In 64% of cases (16/25) ChatGPT-4o and in 60% of cases (15/25) Llama, identified all first-line therapy options considered by the MDT, though with varying priority. ChatGPT-4o presented all the MDT’s first-line therapies in 52% of cases (13/25), while Llama 3 offered a homologous treatment strategy in 48% of cases (12/25). Additionally, both models proposed at least one of the MDT's first-line therapies as their top recommendation in 28% of cases (7/25). The ratings for medical adequacy yielded a mean score of 4.7 (IQR: 4–6) for ChatGPT-4o and 4.3 (IQR: 3–5) for Llama 3. In 17% of the assessments (33/200), MDT members indicated that the LLM recommendations could potentially enhance the MDT's decisions.

**Discussion:**

This study demonstrates the capability of both LLMs to provide viable therapeutic recommendations in ORL head and neck surgery. Llama 3, operating locally, bypasses many data protection issues and shows promise as a clinical tool to support MDT decisions. However at present, LLMs should augment rather than replace human decision-making.

## Introduction

Multidisciplinary (human) tumor boards (MDTs) are a vital component of contemporary cancer treatment, allowing for collaborative decision-making among healthcare specialists from various disciplines. These discussions ensure that patients receive individually tailored treatment plans based on expert opinions [1]. Nevertheless, the pre- and post-processing of MDT is time consuming and may delay treatment. Moreover, geographical factors can also bias decisions as local resources differ [2].

Large Language Models (LLMs) have recently demonstrated the capacity to quickly and efficiently process complex medical data, offering accurate interpretations [3–6]. These models leverage transformer-based architectures, which allow for parallel processing of large amounts of data, making them powerful tools in addressing the limitations of traditional MDTs [7, 8]. LLMs hold the potential to either support or supplement MDT-based tumor management decisions by providing real-time, impartial recommendations based on the latest available medical literature.

Despite these advantages, the use of web-based LLMs in clinical decision-making has been hampered by concerns around data protection and patient confidentiality [9]. This is especially true in medical fields, where sensitive patient data must be handled with the utmost care [10–12]. Locally operated LLMs present a promising alternative, as they function offline, eliminating the need for data transmission over the internet and mitigating the risk of data leakage [13].

Previous studies have explored the potential of LLMs in assisting MDTs [14, 15]. Early evaluations of models like ChatGPT in ORL, head and neck surgery have shown some limitations, including a lack of personalized treatment plans and inconsistency in recommendations [16–18]. These studies were generally based on expert reviews of LLM outputs rather than direct comparisons to MDT recommendations, and they did not address whether LLM-generated information could influence MDT decisions. Furthermore, the reliance on web-based LLMs in prior research has raised concerns about data security, limiting the real-world clinical applicability of these findings.

In this study, we present the first comparison between recommendations made by a standard human MDT in ORL, head and neck surgery and those generated by two LLMs: a web-based model (ChatGPT-4o) and a locally operated model (Llama 3). By addressing the data security concerns of web-based systems, this study seeks to evaluate whether locally run LLMs can offer a secure tool to augment and improve cancer management decision-making in ORL, head, and neck surgery.

## Material and methods

**Fig. 1:**
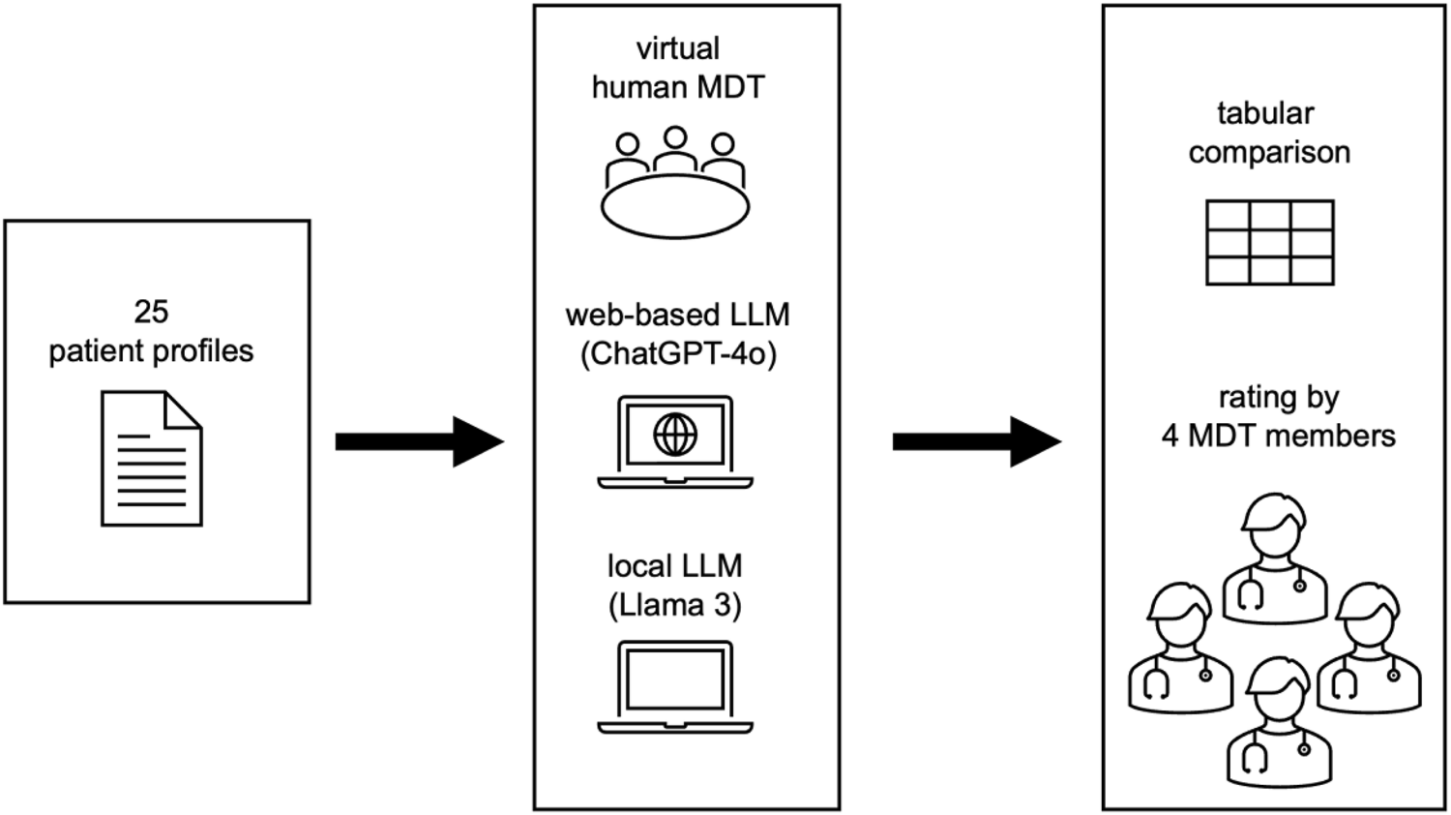
Workflow of the study

The study was performed as follows (Fig. 1). Twenty-five cases resembling patients with a malignant disease from ORL, head and neck surgery were designed (Table 1).

**Table 1:** Constructed cases

	Larynx (L)							
	L1	L2	L3	L4	L5	L6	L7	L8
Age	63	70	65	50	68	66	83	37
Sex	Male	Male	Male	Male	Male	Female	Male	Male
ECOG-score	1	3	1	0	3	1	4	1
Relevant illnesses	Coronary heart disease, stent (age 60), chronic obstructive pulmonary disease, Gold III	Dry alcoholic, liver cirrhosis, Child–Pugh A	Coronary heart disease, myocardial infarction (age 58)	Depression	Arterial hypertension	–	Coronary heart disease; peripheral artery disease (iliac stent insertion 10 years ago); hypertension, atrial fibrillation, heart failure NYHA III, chronic renal failure, oral anticoagulation with Xarelto + ASA	–
Oncological history	–	–	–	–	T3 N2c M0 transoral tumor resection of an oropharyngeal carcinoma, neck dissection on both sides, adjuvant radiation therapy 10 years ago	–	Prostate cancer 2020, transurethral resection of the prostate	-
Smoking	60 pack years	40 pack years	35 pack years	37 pack years	20 pack years	–	50 pack years (stopped 20 years ago)	55 pack years
Drinking	–	Dry	–	–	Active	–	Occasionally	–
Tumor entity	Laryngeal cancer	Laryngeal cancer	Laryngeal cancer	Laryngeal cancer	Laryngeal cancer		Laryngeal cancer	Laryngeal cancer
Localization	**Anterior commissure**	**Supra glottic**	**Anterior commissure/vestibular fold**	**Glottic vocal fold left**	–	–	–	–
First diagnosis	2 month ago	This month	This month	This month	This month	This month	This month	This month
TNM first diagnosis	**cT1b cN0 cM0**	**cT4a cN1 cM0**	**rcT3 cN1 cM0**	**cT1a N0 M0**	**cT4cN2bcM0**	**cT2 cN0 cM0**	**cT3 cN1 cM0**	**cT2 cN0 cM0**
TNM	–	–	–	–	–	–	–	–
Histological subtype	Squamous-cell carcinoma	Squamous-cell carcinoma	Squamous-cell carcinoma	Squamous-cell carcinoma	Squamous-cell carcinoma	Chondrosarcoma	Squamous-cell carcinoma	Squamous-cell carcinoma
Previous therapy and course	Microlaryngoscopy	Emergency tracheostomy, biopsy	Laser resection T2N0M0 (5 months ago) Control panendoscopy (1 week ago)	Microlaryngoscopy	Panendoscopy 1. Vocal fold on the right 2. Pocket fold on the right 3. Arytenoid cartilage on the right. 4. Thyroid cartilage on the right, each with squamous-cell carcinoma	Panendoscopy + biopsy (Arytenoid cartilage left)	Panendoscopy 1st vocal fold right. 2 Right vocal fold, left vocal fold, left vocal fold, Arytenoid cartilage on both sides (all carcinoma positive)	Panendoscopy with biopsy
p16	Negative	Negative	Negative	Positive	Negative	Negative	Negative	Positive
PD-L1	Not determined	TPS 0, CPS 0, IC 0	Not determined	Not determined	Not determined	Not determined	Not determined	Not determined
Grading	Moderately	Poorly	Moderately	Well	Poorly	Moderately	Moderately	Moderately

	Larynx (L)		Nose (N)			Mouth (M)		
	L9	L10	N1	N2	N3	M1	M2	M3
Age	66	55	71	73	85	60	39	63
Sex	Female	Female	Female	Male	Male	Female	Male	Male
ECOG-score	2	1	1	0	1	0	0	1
Relevant illnesses	Arterial hypertension; diabetes mellitus type II, chronic obstructive pulmonary disease: gold III	Depression, fibromyalgia	Arterial hypertension	–	Arterial hypertension	Arterial hypertension	–	Arterial hypertension, diabetes mellitus type II, polyneuropathy, benign prostatic hyperplasia
Oncological history	–	–	–	–	–	–	–	–
Smoking	80 pack years		–	–	–	40 pack years	–	55 pack years
Drinking	–	–	–	–	–	Dry	–	Active
Tumor entity	Laryngeal cancer	Laryngeal cancer	Nasal cancer	Nasal cancer	Nasal cancer	Tongue cancer	Tongue cancer	Oral Cancer
Localization	–	–	**Septum left**	**Right ethmoid bone**	**Right maxillary sinus**	**Left tongue edge**	**Left tongue edge**	**Mouth bottom left (crossing the midline)**
First diagnosis	6 years ago	This month	1 year ago	This year	3 years ago	This year	This month	this month
TNM first diagnosis	**cT1a cN0 cM0**	**Cacinoma in situ left**	**cT2N0M0**	**cT4a cN0 cM0**		**cT1cN0cM0**	**T1N0**	**cT4a cN2c cM0**
TNM	**rcT3 cN2b cM1 (pulmonary singular focus)**	–	–	–	**rcT1cN0cMx**	–	**pT3, pN3b(23/69, ECE +), L1, V0, Pn1, R0**	**cT4a cN2c cM0**
Histological subtype	Squamous-cell carcinoma	Cacinoma in situ	Squamous-cell carcinoma	Squamous-cell carcinoma after inverted papilloma	Squamous-cell carcinoma	Squamous-cell carcinoma	Squamous-cell carcinoma	Squamous-cell carcinoma
Previous therapy and course	pT1a cN0 cM0 vocal fold carcinoma, left chordectomy 6 years ago Control panendoscopy (evidence of carcinoma in situ and renewed laser resection alio loco 5 years ago), continued nicotine consumption, tumor follow-up irregularly observed Panendoscopy 1st vocal fold on the right, vocal fold on the left, pocket fold on the left, arytenoid hump on both sides (all carcinoma positive)	Panendoscopy, vocal fold stripping	Paranasal sinus revision on both sides Paranasal sinus mapping on both sides: tumor nasal septum left remaining biopsies tumor-free	Paranasal sinus revision on both sides Paranasal sinus mapping on both sides	Paranasal sinus surgery Caldwell Luc surgery and endonasal paranasal sinus surgery Revision paranasal sinus surgery: Squamous-cell carcinoma	Biopsy	Panendoscopy and biopsy transoral tumor resection, neck dissection on both sides, radial flap	Panendoscopy and biopsy
p16	Negative	Not determined	Negative	Negative	Positive	Negative	Negative	Negative
PD-L1	TPS 50, CPS 60, IC 10	Not determined	Not determined	Not determined	Not determined	Not determined	Not determined	TPS 90, CPS 100, IC 10
Grading	Moderately	Not determined	Well	Moderately	Poorly	Moderately	Moderately	Moderately

	Pharynx (P)					Cancer of unknown primary (CUP)	Craniomaxillofacial surgery (CMFS)		
	P1	P2	P3	P4	P5	CUP1	CMFS1	CMFS2	CMFS3
Age	60	63	81	45	72	40	64	60	50
Sex	Male	Female	Male	Male	Female	Female	Male	Male	Male
ECOG-score	3	0	1	0	1	0	1	1	2
Relevant illnesses	Chronic obstructive pulmonary disease	–	Hypothyroidism, arterial hypertension, chronic obstructive pulmonary disease: gold II, coronary heart disease, peripheral arterial disease, left thigh (stent insertion)	–	Arterial hypertension, diabetes mellitus type II, adipositas per magna	–	Arterial hypertension, alcoholic, obesity	Arterial hypertension, Diabetes mellitus type II, adipositas	Arterial hypertension, adipositas, benign prostatic hyperplasia
Oncological history	Chordectomy: squamous-cell carcinoma vocal fold left Panendoscopy with recurrence Laryngectom, neck dissection on both sides, tracheotomy	–	pTA low grade urothel carcinoma (Transurethral resection of the urinary bladder)	–	pT2, pN0 Tonsil carcinoma right Tumor resection + neck dissection radial flap + right 2014 Ductal carcinoma in situ Surgery + sentinel (–)	–	–	–	–
Smoking	48 pack years	–	50 pack years	–	60 pack years	–	30 pack years	40 pack years	20 pack years
Drinking	–	–	Active	–	–	–	Active	Active	–
Tumor entity	Tongue cancer left	Oropharynx cancer	Hypopharynx cancer	Tonsil cancer	–	Cancer of unknown primary	Cancer Mouth bottom	Mandibula cancer	Squamous-cell carcinoma
Localization	**Base of tongue left**	**Oropharynx: soft palate left with infiltration in nasopharynx**	**right**	**Tonsil left**	**Right tongue edge**	**Lymph node metastasis on the left**	**Mouth bottom right**	**Left mandible, tongue infiltration, midline crossing**	**Regio 16 17**
First diagnosis	This month	5 years ago	This month	This month	10 years ago	This month	2 years ago	This week	This month
TNM first diagnosis	**cT2 cN0 cM0 p16-**	**cT3N2 p16 + **	**cT3 cN3 cM0**	**cTX cN1 cM0**	**pT2 pN0 cM0**	**cTX N2b cM0**	**cT1 cN1 M0**	**cT4 cN3b cM1 (pulmonary, bilateral foci do not match lung cancer, multiple foci)**	**cT1 cN0 cM0**
TNM	–	**rT4a, N1, L1, V0, Pn0, R1, M0**	–	**pT1pN1cM0**	**cT4 cN2 cM1 (pulmonal multiple), p16-**	**cTX N2b cM0**	–	–	–
Histological subtype	Squamous-cell carcinoma	Squamous-cell carcinoma	–	Squamous-cell carcinoma	Squamous-cell carcinoma	Squamous-cell carcinoma	Squamous-cell carcinoma	Squamous-cell carcinoma	Squamous-cell carcinoma
Previous therapy and course	Panendoscopy and biopsy	Primary chemoradioation 5 years ago	–	Lymph node exstripation 01/2024 Cancer of unknown primary, panendocopy + tonsillectomy: squamous-cell carcinoma Tonsil 02/2024	–	Lymph node extirpation level II (for suspected lymphoma)	Biopsy one week ago	Biopsy one week ago	Leukoplakia for 2 years. Biopsy 2 weeks ago: squamous-cell carcinoma
p16	Negative	Positive	–	Positive	Negative	Positive	Not determined	Not determined	Negative
PD-L1	Not determined	TPS 0, CPS 0, IC 0	–	Not determined	Not determined	Not determined	Not determined	Not determined	Not determined
Grading	Moderately	Moderately	–	Moderately	Moderately	Moderately	Well	Poorly	Moderately

The bold font indicate important information

Profiles were correlated with realistic clinical scenarios from the author's clinical experience and reviewed by four experienced senior consultants. Each case was prepared in the same semantic fashion including age, sex, ECOG stadium, medical history, oncological history, drug consumption (cigarettes, alcohol), tumor entity, localization, first diagnosis, TNM-stadium, histological type, p16-, PD-L1 status, grading and treatment history to date. Each case was then presented in a multidisciplinary MDT. The implemented MDT included an otorhinolaryngologist, maxillofacial surgeon, radiation oncologist, medical oncologist, radiologist and a pathologist. MDT members were chosen from different affiliations in order to reduce geographical bias. In a virtually held MDT consultation each case was discussed and therapeutic regimen was recommended.

**Fig. 2:**
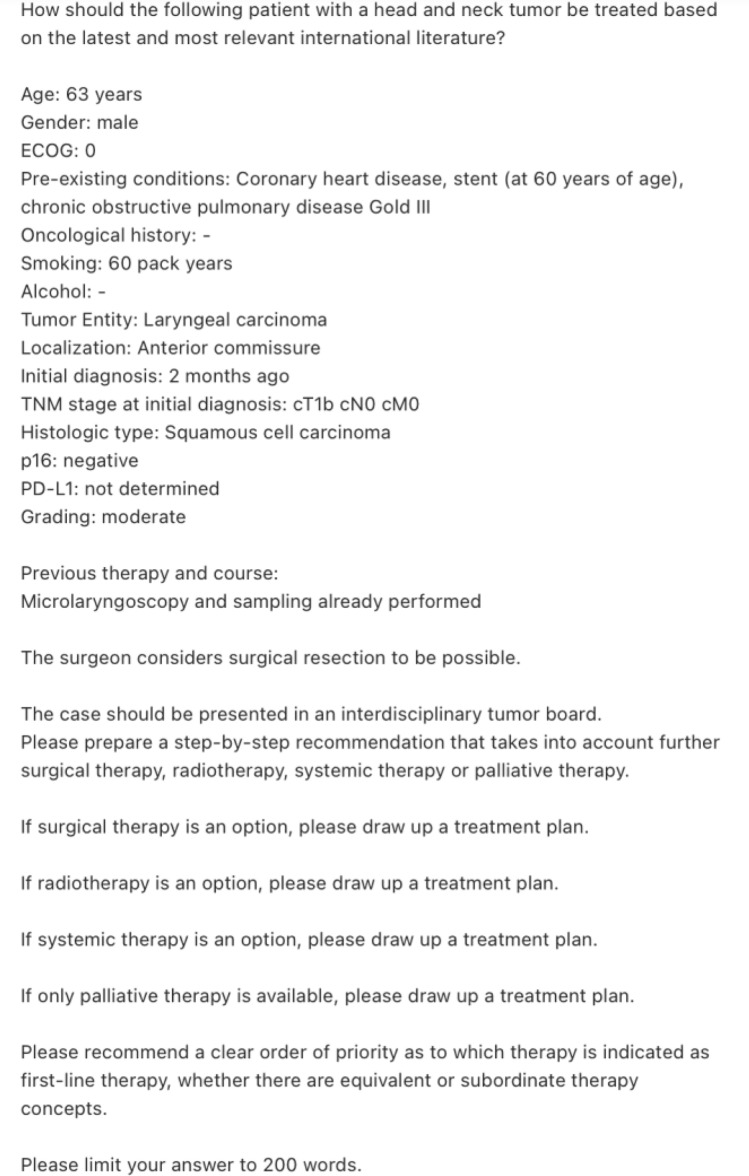
Exemplary prompt for patient (L1) translated by deepL.com (Cologne, Germany)

Subsequently, each individual tumor case was passed to two different LLMs. Different prompting strategies inspired by Griewing et al. were tried, to exclude biased output [15]. The finally implemented prompt also stated the patient's details including age, sex, ECOG stadium, medical history, oncological history, drug consumption (cigarettes, alcohol), tumor entity, localization, first diagnosis, TNM-stadium, histological type, p16-, PD-L1 status, grading and therapy to date. If applicable, the prompt also mentioned the surgeon's opinion on resection of the tumor, before mentioning that the patient is presented in an MDT and referring to surgical-, radio-, systemic- and palliative therapy options and asking for a treatment plan for each entity if applicable. Moreover, the prompt required a clear ranking of suitable treatments. Finally, the LLMs were forced to limit the answer to 200 words (Fig. 2). After designing the prompt, patient profiles were passed to the latest web-based version of OpenAI’s ChatGPT-4o and the locally running Llama 3 LLM without further training. The local LLM was run on a standard MacBook Pro 2023, M3 Pro, 18 GB RAM (Apple, California, USA).

Recommendations of the human tumor board, web-based ChatGPT-4o and locally Llama 3 were accumulated in an Excel sheet to allow a side-by-side comparison and analyzing concordance. Moreover, 4 members of the MDT rated recommendations provided by ChatGPT-4o and Llama 3 for medical adequacy on a 6-point Likert-scale (0 = very poor, 6 = excellent). Furthermore, the 4 raters stated if, in their opinion, the information provided by the LLM might have influenced the recommendation of the MDT (yes/no/abstention). Moreover, there was space for raters to express free text comments.

Statistical analysis of Likert-scale ordinal variables (qualitative-ordinal) was performed with GraphPad Prism for MacOS Version 10.3.0 (GraphPad Software, La Jolla, CA, USA). Normality distribution was tested with the D’Agostino and Pearson test. Group comparisons were performed using the Mann Whitney Test. Ethics approval was not required as no real patient data was processed.

## Results

An overview of the therapy recommendations as stated by the MDT, ChatGPT-4o and Llama 3 as well as their recommendation on curative or palliative therapy regimen and agreement in first-line therapy regimen for each specific case are shown in Table 2.

**Table 2:**
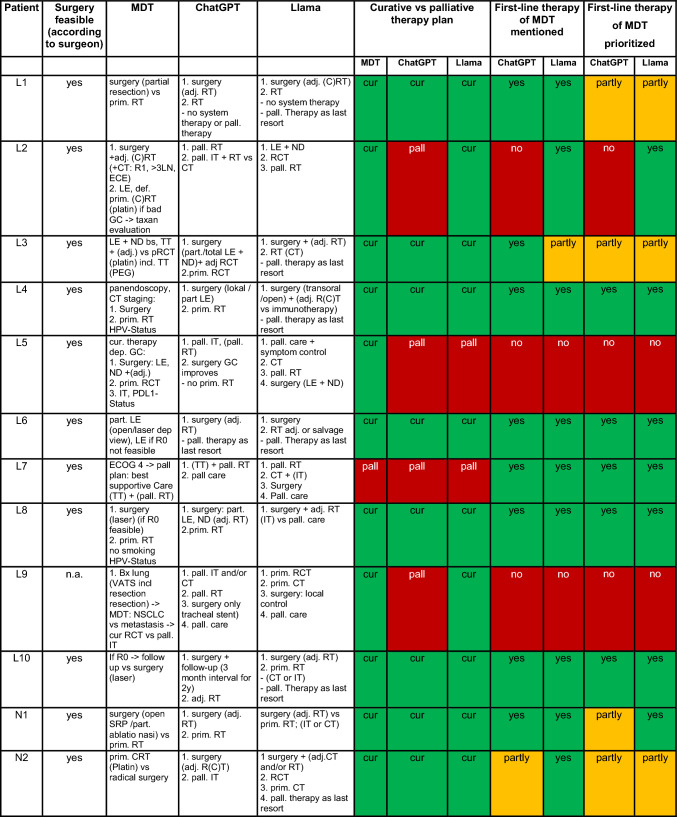

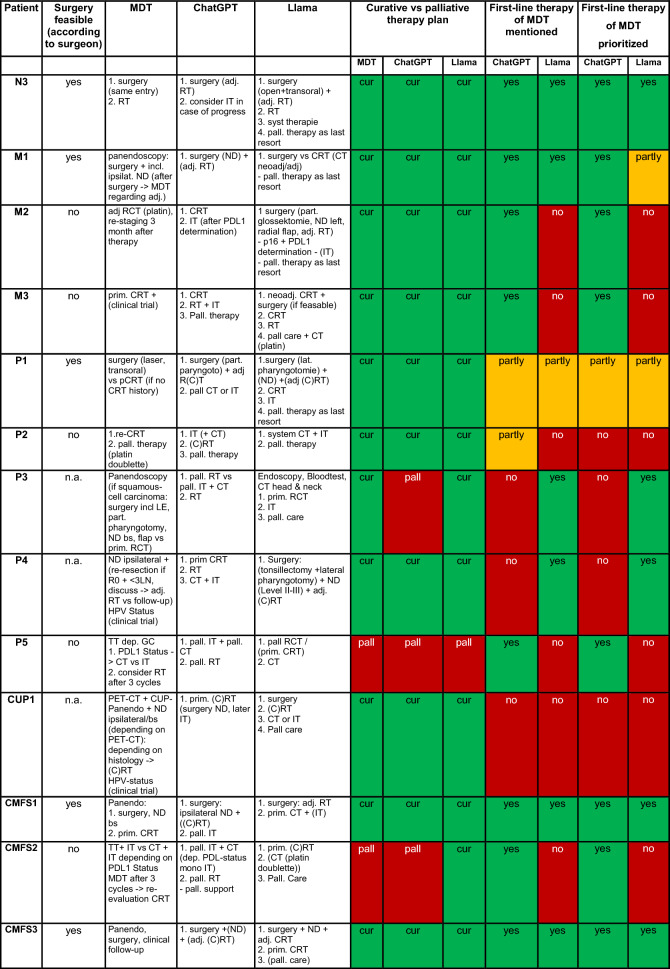
Therapy recommendations of MDT, ChatGPT-4o and Llama 3

*MDT* (human) multidisciplinary tumor board recommendations; *prim* primary; *adj* adjuvant; *pall*. palliative; *cur* curative; *RT* radiation therapy; *CRT* chemoradiation therapy; *CT* chemotherapy; *IT* immunotherapy; *R* resection margin; *LN* lymph node; *ECE* extra capsular expansion; *GC* general condition; *LE* laryngectomy; *ND* neck dissection; *bs* both sides; *TT* tracheotomy; *PEG* percutaneous endoscopic gastrostomy; *CT* computerized tomography; *HPV* humane papillomavirus; *ECOG-Status* Eastern Cooperative Oncology Group-Status; *Bx* Biopsy; *VATS* video-assisted thoracic surgery; *SRP* septorhinoplastic

The color increases readability substantially

Table 3 summarizes the comparison between the MDT, ChatGPT-4o and Llama 3. The human tumor board suggested a curative therapy regimen for 88% (22/25), ChatGPT-4o for 72% (18/25) and Llama 3 88% (22/25) of the cases. Thus, a palliative strategy was stated for 12% (3/25) of the cases by the human tumor board and Llama 3, while ChatGPT-4o suggested a palliative scenario 28% (7/25). ChatGPT-4o and the human tumor board showed a concordance for this aspect of 84% (21/25). In all cases of discordance ChatGPT-4o favored a palliative therapy whereas the human board suggested a curative therapy regimen. Llama 3 showed a concordance of 92% (23/25) with the human board.

**Table 3:** Summarized comparison of therapy recommendation

Curative vs palliative therapy regimen					
	MDT	ChatGPT-4o		Llama 3	
			Concordance with MDT		Concordance with MDT
Curative therapy regimen	22 patients (22/25 = 88%)	18 patients (18/25 = 72%)	84% (21/25)	22 patients (22/25 = 88%)	92% (23/25)
Palliative therapy regimen	3 patients (3/25 = 12%)	7 patients (7/25 = 28%)		3 patients (3/25 = 12%)	

First-line therapy of the MDT mentioned by the LLMs		
	ChatGPT-4o	Llama 3
Stated all first-line therapy regimen of the MDT	16 patients (16/25 = 64%)	15 patients (15/25 = 60%)
Stated some first-line therapy regimen of the MDT as first-line	5 patients (5/25 = 20%)	5 patients (5/25 = 20%)
Stated all first-line therapy regimen stated as first-line like the MDT	13 patients (13/25 = 52%)	12 patients (12/25 = 48%)

First-line therapy regimen stated			
	MDT	ChatGPT-4o	Llama 3
First-line therapy regimen			
Surgery	12 patients (12/25 = 48%)	13 patients (13/25 = 52%)	16 patients (16/25 = 64%)
Radiotherapy	–	3 patients (3/25 = 12%)	1 patient (1/25 = 4%)
Chemoradiotherapy	3 patients (3/25 = 12%)	3 patients (3/25 = 12%)	2 patients (2/25 = 8%)
(Chemo)radiotherapy	–	1 patient (1/25 = 4%)	1 patient (1/25 = 4%)
Surgery vs. radiotherapy (equal)	2 patients (2/25 = 8%)	–	1 patient (1/25 = 4%)
Surgery vs. chemoradiotherapy (equal)	3 patients (3/25 = 12%)	–	1 patient (1/25 = 4%)
Immune- and/or chemotherapy	2 patients (2/25 = 8%)	5 patients (5/25 = 20%)	1 patient (1/25 = 4%)
Further DIAGNOSTIC	2 patients (2/25 = 8%)	–	1 patient (1/25 = 4%)
Best supportive care	1 patient (1/25 = 4%)	–	1 patient (1/25 = 4%)

Therapy regimen considered beyond first-line					
	MDT	ChatGPT-4o		Llama 3	
Therapy regimen considered beyond first-line			Concordance with MDT		Concordance with MDT
Surgery	16 patients (16/25 = 64%)	14 patients (14/25 = 56%)	84% (21/25)	20 patients (20/25 = 80%)	76% (19/25)
Radiotherapy	10 (patients 10/25 = 40%)	21 patients (21/25 = 84%)	56% (14/25)	19 patients (19/25 = 76%)	56% (14/25)
Chemoradiotherapy	12 (patients 12/25 = 48%)	12 patients (12/25 = 48%)	76% (19/25)	14 patients (14/25 = 56%)	56% (14/25)
Chemotherapy	4 (patients 4/25 = 16%)	10 patients (10/25 = 40%)	76% (19/25)	13 patients (13/25 = 52%)	52% (13/25)
Immunotherapy	4 (patients 4 = 25 = 16%)	18 patients (18/25 = 72%)	44% (11/25)	13 patients (13/25 = 52%)	40% (10/25)

In 64% (16/25) of the instances ChatGPT-4o and in 60% of cases (15/25) Llama 3 mentioned all first-line recommendation provided by the human board as a possible therapy regimen, although sometimes in a different order. Whereas ChatGPT-4o stated at least one of the first-line recommendations in another 12% (3/25) of the instances, Llama 3 did so in 8% (2/25) more instances. While ChatGPT-4o stated all first-line therapy regimen as stated by the human board in 52% (13/25), Llama 3 showed this high concordance in 48% (12/25). Both, ChatGPT-4o and Llama 3 stated at least one of the human board´s first-line therapy regimen as first-line regimen in 20% (5/25) of the cases each.

**Fig. 3:**
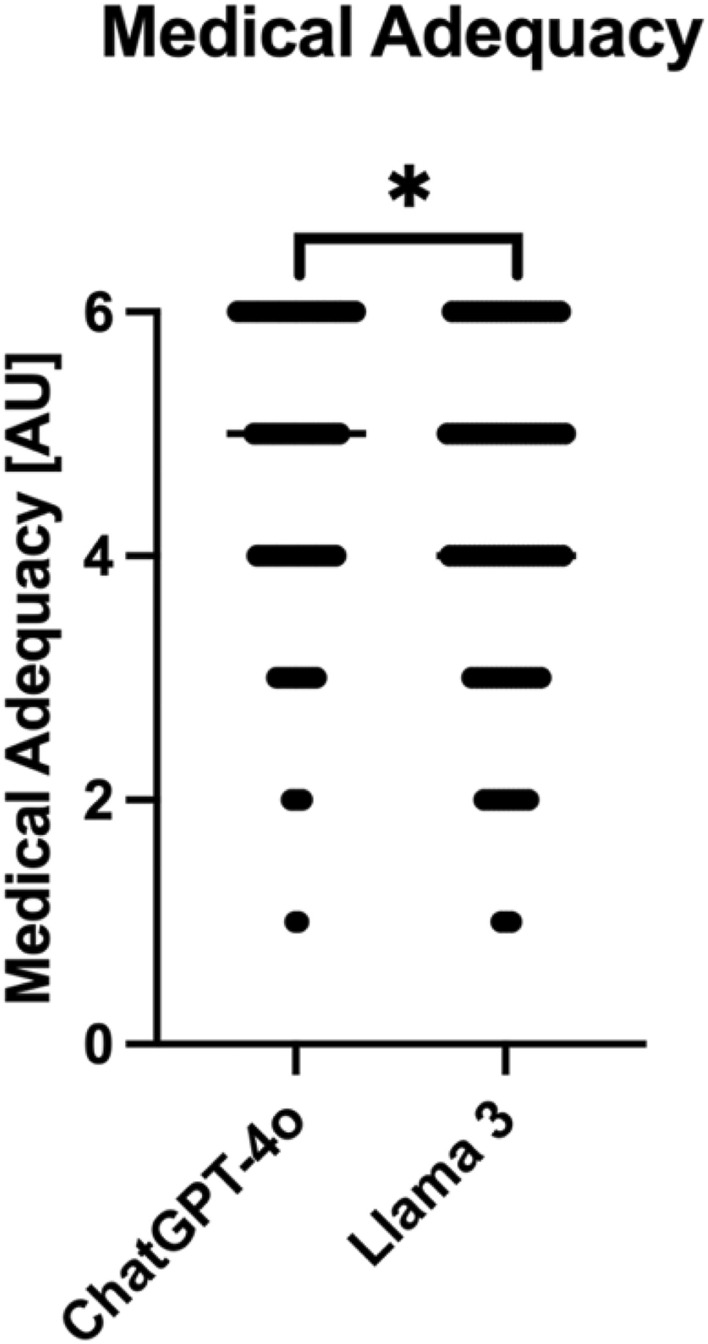
Medical Adequacy for recommendations stated by ChatGPT-4o (mean 4.7; range 1–6) and Llama 3 (mean 4.3; range 1–6) as rated by the 4 MDT members. Data shown as a scatter dot blot with each point resembling an absolute value (bar width resembling a high amount of individual values).  Normality distribution was tested with the D’Agostino and Pearson test. Group comparisons were performed using the Mann Whitney Test. *p < 0.05

The rating of 4 MDT revealed a mean score of 4.7 (IQR 4–6) for ChatGPT-4o and a significant inferior (p < 0.001) mean score of 4.3 (IQR 3–5) for Llama 3 on medical adequacy (Fig. 3). For one patient, two raters concluded that the additional information from the LLM might have influenced the MDT decision. In this case, the patient had ECOG stage 3 and the MDT had suggested a curative treatment regimen. Overall, the raters stated that the information from the LLM could have influenced the decision of the tumor board in 17% (33/200) of the cases. However, this statement varied beyond raters. One rater stated that the information from the LLMs may have influenced the decision in for 32% (8/25) of the cases for ChatGPT-4o and Llama 3, respectively. In contrast, another rater saw no possible influence on the MDT decision in any case.

Whereas the human tumor board usually provided detailed information about the surgery, the LLMs often recommend surgical therapy without specification or the extent of surgery. Although web-based ChatGPT-4o and locally run Llama 3 received exactly the same prompts, ChatGPT-4o responded in German whereas Llama 3 answered in English language. While ChatGPT-4o met the word limit every single time (mean 150 words), Llama 3 provided too long answers and met the word limit just once (mean 246 words). Once Llama 3 pretended to “accept” the word limit by providing “Word count: 199” at the end of the response, in this case the response was 248 word long.

## Discussion

Previous monocentric studies evaluated the feasibility of LLMs in providing recommendations for tumor treatment regimes in ORL, head and neck surgery [16–18]. However, most of these studies were dedicated to older versions and mainly focused solely on ChatGPT, with one exception evaluating Claude 3.

Our study however is the first one evaluating the web-based ChatGPT-4o and, in order to address data protection queries, the locally run LLM (Llama 3). In contrast to prior studies, recommendations of the LLMs were directly compared to human tumor board decisions and subsequently rated by experts. More specific, four MDT members evaluated the LLMs' recommendations for medical adequacy using a 6-point Likert scale and assessed whether the provided information could have influenced the MDT's final decision.

Our findings reveal high concordance between the LLMs and the MDT, particularly in the critical distinction between curative and palliative therapy strategies. Llama 3, the locally run model, exhibited a 92% (23/25) concordance rate with the MDT, while ChatGPT-4o achieved 84% (21/25). ChatGPT-4o mentioned all first-line recommendation of the MDT in 64% (16/25) of the patients, Llama in 60% (15/25). While ChatGPT-4o stated the same first-line recommendations as the MDT as first-line in 52% (13/25) of the cases, Llama 3 did so in 48% (12/25). Whereas ChatGPT-4o stated the first-line recommendation of the MDT in parts as first-line in 12% (3/25) more cases, Llama 3 achieved the same in 8% (2/25) more cases. Accordingly, in 72% (18/25) ChatGPT-4o and in 68% (17/25) of the cases Llama 3 stated at least one identical MDT first-line therapy regimen as first-line. This competence of the current LLMs both on and off-line is surprisingly sufficient, especially when considering that previous studies in ORL were more negative regarding individualized therapy recommendations of LLMs [16, 17].

First-line surgery as sole therapy was most frequently suggested by the MDT and LLMs alike, with the MDT for 48% (12/25), ChatGPT-4o for 52% (13/25) and Lama for 64% (16/25) of the cases. While the MDT provided a more detailed surgical therapy plan i.e. “local resection and neck dissection ipsilateral” the LLMs frequently lack specification of the surgery. This is particularly problematic if the LLM, like Llama did in an exemplary case (M1), concentrates on the resection of the primary tumor and does not mention a necessary neck dissection.

To provide a realistic setting, we intentionally implemented some obstacles into the patient profiles to provide a more realistic setup of flawed and/or misleading submissions to the MDT. In one case a patient with cT3 cN3 cM0 Hypopharynx Carcinoma (P3) without any actual histopathological confirmation, both ChatGPT-4o and Llama 3 suggested treatment plans whereas the MDT recommended a panendoscopy with subsequent biopsy first. In this case, ChatGPT-4o suggested a palliative therapy for a tumor which was not histologically proven at that time. Llama 3 recommended primary chemoradiotherapy as first line therapy although neither histological evaluation nor a surgeon's statement regarding resectability was apparent.

A further pitfall for the LLMs was a cancer of unknown primary (CUP) Syndrome (CUP1). Here none of the LLMs suggested a CUP panendoscopy or a PET CT like the MDT. In addition, Llama did not realize that the primary tumor localisation is in fact unknown and therefore its resection is not feasible.

Furthermore, in one instance (M2) Llama did not realize that the patient had already undergone surgery with curative intent and that the presentation was only for adjuvant therapy. Here, one can argue that the prompt should have been adapted, as MDT submissions usually also provide information about a pre- or post-therapeutic presentation. However, the simulated case represents a scenario of an imperfect submission, which unfortunately does occur in everyday clinical practice.

Moreover, we noticed that Llama 3 ignored the German input by answering in English instead of German all the time. The same happened with regards to the word limit, not only did Llama 3 exceed the word limit nearly every time, in one case Llama 3 even claimed to respect the word limit even though it was exceeded. These examples suggest that LLMs tend to ignore instruction if they are not capable of fulfilling them, rather than admitting deficiencies. Especially for tumor treatment plans this is hazardous and should be considered when consulting LLMs in these matters. This fact underlines the inference of previous studies (in ORL and beyond) that LLMs are very promising for augmenting MDT but cannot and should not replace them [14–17].

Despite some fundamental flaws, the LLMs received respectable ratings for medical adequacy on a 6-point Likert-scale by our 4 evaluating MDT members visualized by a mean score of 4.7 (IQR 4–6) for ChatGPT-4o and 4.3 (IQR 3–5) for Llama 3 (Fig. 3). This might be explained by the fact that allegedly less important facts, such as ECOG status or smoking history, can easily fade into the background in a MDT whilst they were frequently highlighted by the LLMs. In our study, the MDT had suggested a curative treatment regimen for a patient with ECOG stage 3 (L2). Here, two raters stated that the information from the LLM might have changed the recommendation of the MDT. Raters found it positive that the LLMs highlighted chronic diseases of the patients and addiction history. Overall, raters stated in 17% (33/150) of the ratings, that the information of the LLM might have changed the recommendation of the board. In individual cases, the use of LLMs can therefore potentially have a very positive influence on the fate of a patient. Particularly when considering the fact that the resources for an MDT can vary enormously depending on the environment of MDTs [2].

Obviously, this study has some limitations, including the fact that constructed cases instead of real patient profiles were used. However, even after anonymization of data sets, entry of real cases into a web-based LLM was not approved by our data protection officer. As a comparison of the web-based LLM ChatGPT-4o and the locally run LLM Llama 3 was a key objective of this study we were limited to constructed patient profiles. Moreover, one could argue that a MDT resembling members from different affiliations is no realistic scenario, however, we choose this design by default in order to limit subjective and institutional biases within the MDT decision. In fact, we noticed local differences in default treatment regimens, for instance whereas in one institution a dental renovation is implemented prior to radiation therapy, other institutions waive the step in order to accelerate the start of the treatment. This emphasizes exemplary the virtue of a multicentric approach.

Taking these limitations into account, the present study is the first study evaluating a locally run LLM (Llama 3) on ORL, head and neck surgery MDT recommendations. Interestingly, the results of the local LLM (Llama 3) were inferior to the web-based ChatGPT-4o. With regard to the decision on a curative or palliative procedure, the agreement between the MDT and the local Llama 3 LLM was even higher. Since this is the first study in ORL, head and neck surgery evaluating a local LLM, the potential for optimization is certainly not exhausted yet. First, newer versions and the comparison of different local LLMs might improve results. Secondly, special training on specific ORL, head and neck literature and aligned prompting might further improve the outcome. It is therefore likely that local LLMs will catch up with the web-based versions in the near future. Especially in medicine, passing highly sensitive private patient data to privately owned, web-based LLMs is hardly imaginable in clinical practice [9]. Despite data privacy, the open-access structure of local LLMs is quite cost-effective requiring just a sole standard computer available for less than €1000. Furthermore, local LLMs provide the opportunity of deployment in remote regions without internet connection. Thus, these models represent also a great opportunity for low-resource settings in low and middle income countries (LMICs). Future research regarding medical application should therefore focus on local LLMs.

With regards to the local and open-source character of Llama 3, the results of this study might reflect an important step to actual implementation of locally run, data protection compliant LLMs into real clinical practice. Despite our promising findings, it is unlikely that MDTs are close to being replaced by LLMs. Human assessment in tumor board decision-making currently proves more reliable than LLM-driven assessments, especially as they will be reluctant to put any decision making regarding their therapeutic faith in the hands of a computer [19]. Additionally, the medicolegal implications are entirely unclear at this point. Rather MDTs are likely to be augmented by locally run LLMs helping to reduce geographical bias of tumor boards [2, 20]. Future studies should thus evaluate the integration of (local) LLMs in the MDT process.

## Data Availability

The relevant data to support the results are included in the manuscript.
